# Supplementary material for: Space radiation damage rescued by inhibition of key spaceflight associated miRNAs
Source: Nat Commun. 2024 Jun 11;15:4825. doi: 10.1038/s41467-024-48920-y (PMC11166944; doi:10.1038/s41467-024-48920-y)
Supplement: Supplementary file 1 — Supplementary Information [file 41467_2024_48920_MOESM1_ESM.pdf]

Supplementary Information

Space Radiation Damage Rescued by Inhibition of Key Spaceflight Associated miRNAs

J. Tyson McDonald, JangKeun Kim, Lily Farmerie, Meghan L. Johnson, Nidia S. Trovao, Shehbeel Arif, Keith Siew, Sergey Tsoy, Yaron Bram, Jiwoon Park, Eliah Overbey, Krista Ryon, Jeffrey Haltom, Urminder Singh, Francisco J. Enguita, Victoria Zaksas, Joseph W. Guarnieri, Michael Topper, Douglas C. Wallace, Cem Meydan, Stephen Baylin, Robert Meller, Masafumi Muratani, D. Marshall Porterfield, Brett Kaufman, Marcelo A. Mori, Stephen B. Walsh, Dominique Sigaudo-Roussel, Mebarek Saida, Massimo Bottini, Christophe A. Marquette, Eve Syrkin Wurtele, Robert E. Schwartz, Diego Galeano, Christopher E. Mason, Peter Grabham, Afshin Beheshti

Supplementary Figure 1. Quality Checks for the WGCNA analysis on the miRNA-seq data.
Supplementary Figure 2. Co-expressed miRNA network heatmaps from the WGCNA analysis.
Supplementary Figure 3. Global transcriptomic analysis reveals that antagomirs revert gene profiles closer to control samples (with adj. *p*-values).
Supplementary Figure 4. Orphan and EB gene analysis on the RNA-seq analysis from the 3D mature and angiogenesis cell culture models.
Supplementary Figure 5. Pathway analysis on the 21 key genes restored to basal level after antagomir treatment.
Supplementary Figure 6. Clustering of the 21 key genes from scRNA-sequence data from the i4 astronauts.
Supplementary Figure 7. The interaction of the three miRNAs with the 21 gene targets from the NASA Twin Study for all cell types.
Supplementary Figure 8. Suppression of senescence related pathways and genes after irradiation with GCR.

Supplementary Table 1. Experimental validation for the gene targets to miR-16-5p, miR-125b-5p. and let-7a-5p from Fig. 5.

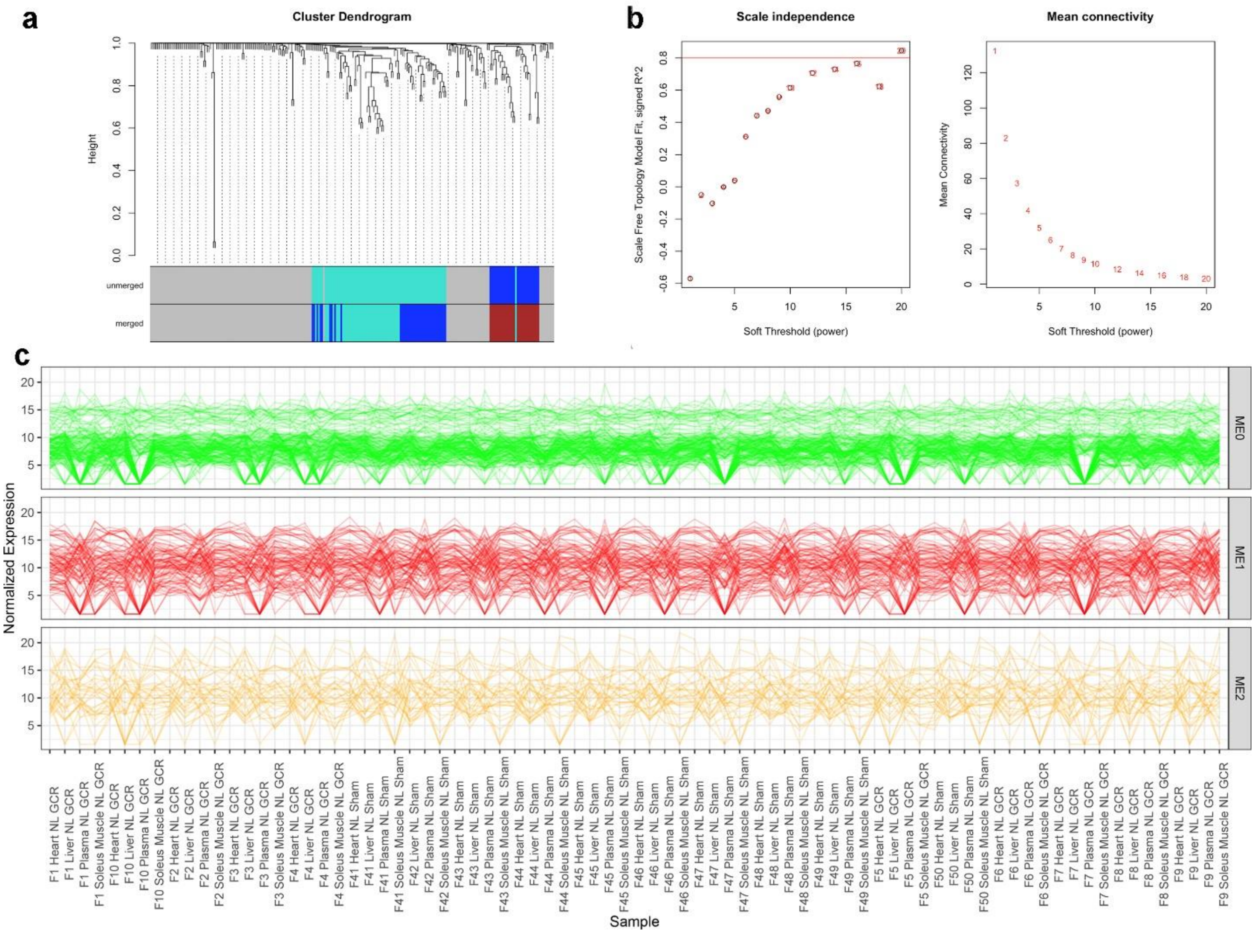

Supplementary Figure 1. Quality Checks for the WGCNA analysis on the miRNA-seq data. a) A cluster dendrogram for the grouping of the miRNAs part of the three network modules that emerged from the analysis. **b)** The Scale Independence plot displaying an R2 cut-off of 0.8 (left plot) and the Mean Connectivity plot (right plot), where a soft threshold of 16 was used to prune the miRNA network. **c)** The line plot displaying normalized expression values for the miRNA-seq data for the individual samples in the analysis across the three miRNA network modules.

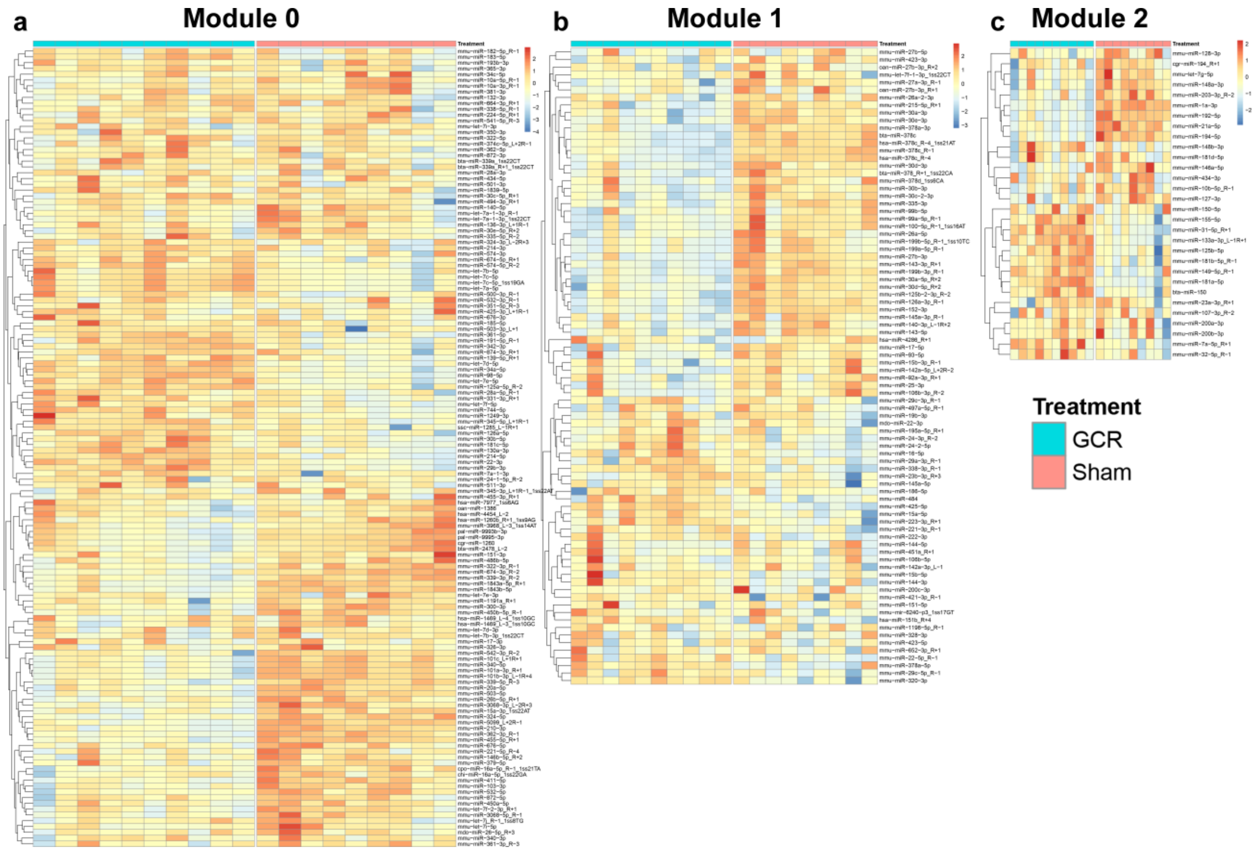

Supplementary Figure 2. Co-expressed miRNA network heatmaps from the WGCNA analysis. Heatmaps displaying the normalized expression of individual miRNAs part of **a)** Module 0, **b)** Module 1, and **c)** Module 2. The heatmap columns display the mice samples split by GCR and Sham treatment.

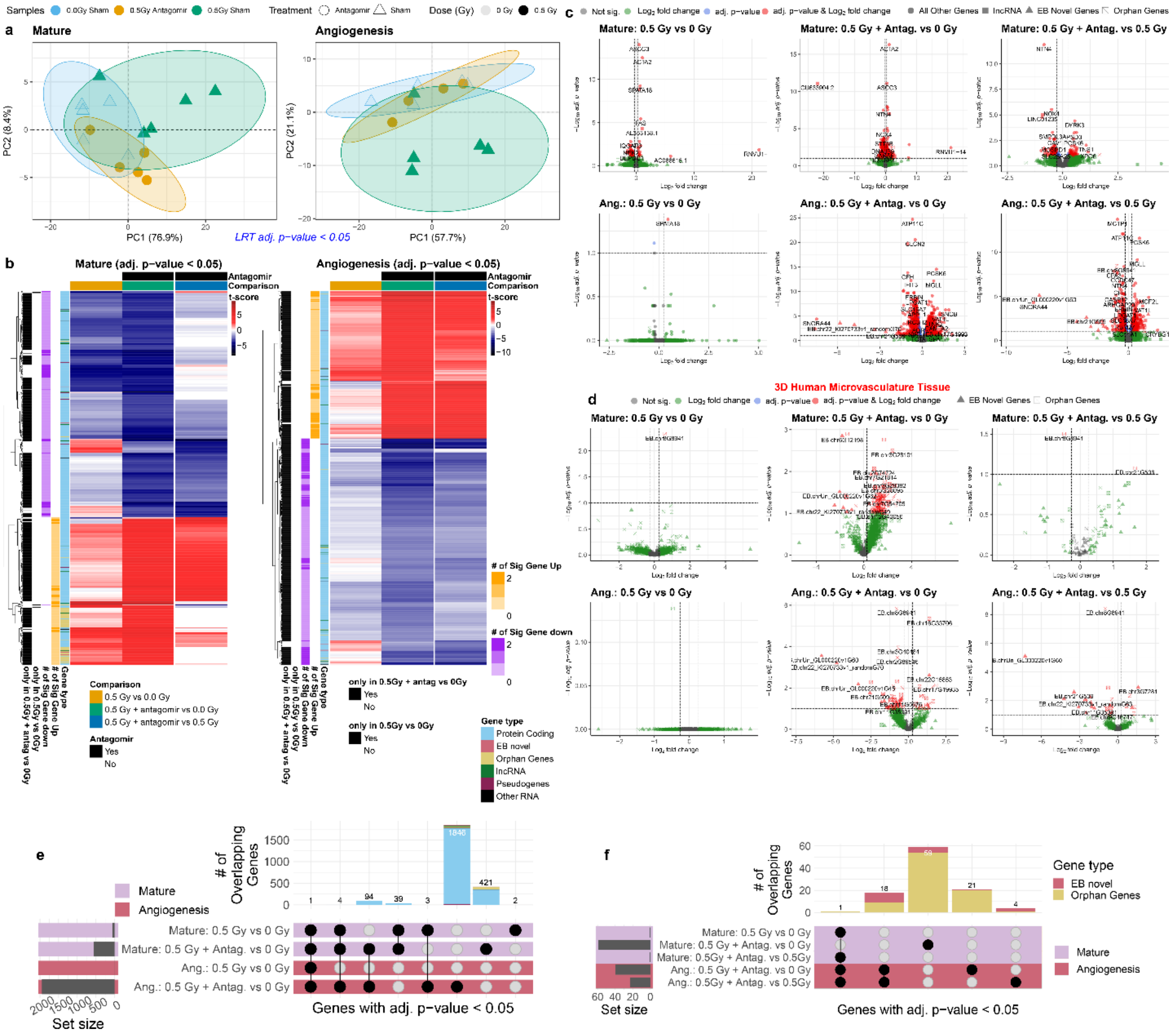

Supplementary Figure 3. Global transcriptomic analysis reveals that antagomirs revert gene profiles closer to control samples (with adj. *p*-values). a) Principal Component Analysis (PCA) of the significantly regulated genes (adj. *p*-value < 0.05) for all conditions compared with likelihood ratio test (LTR) analysis for both mature and angiogenesis 3D microvessel cell culture models. b**)** Heatmap of significantly regulated genes (adj. *p*-value < 0.05) for mature and angiogenesis cell culture models. For each gene (*i.e.* row), the criteria for display was to have at least one comparison per gene to be significantly regulated. Then the trends for non-significant genes for that row were also displayed. The log_2(fold-change) values are displayed. The side color bars indicate the number of significant genes that are either up- or down-regulated per row and also the type of gene. Volcano plots for **c)** all genes and **d)** only orphan and EB genes. Upset plots displaying the overlapping significantly regulated genes (adj. *p*-value < 0.05) for mature and angiogenesis models with and without antagomir treatment for **e)** all the gene types and **f)** only orphan and EB genes.

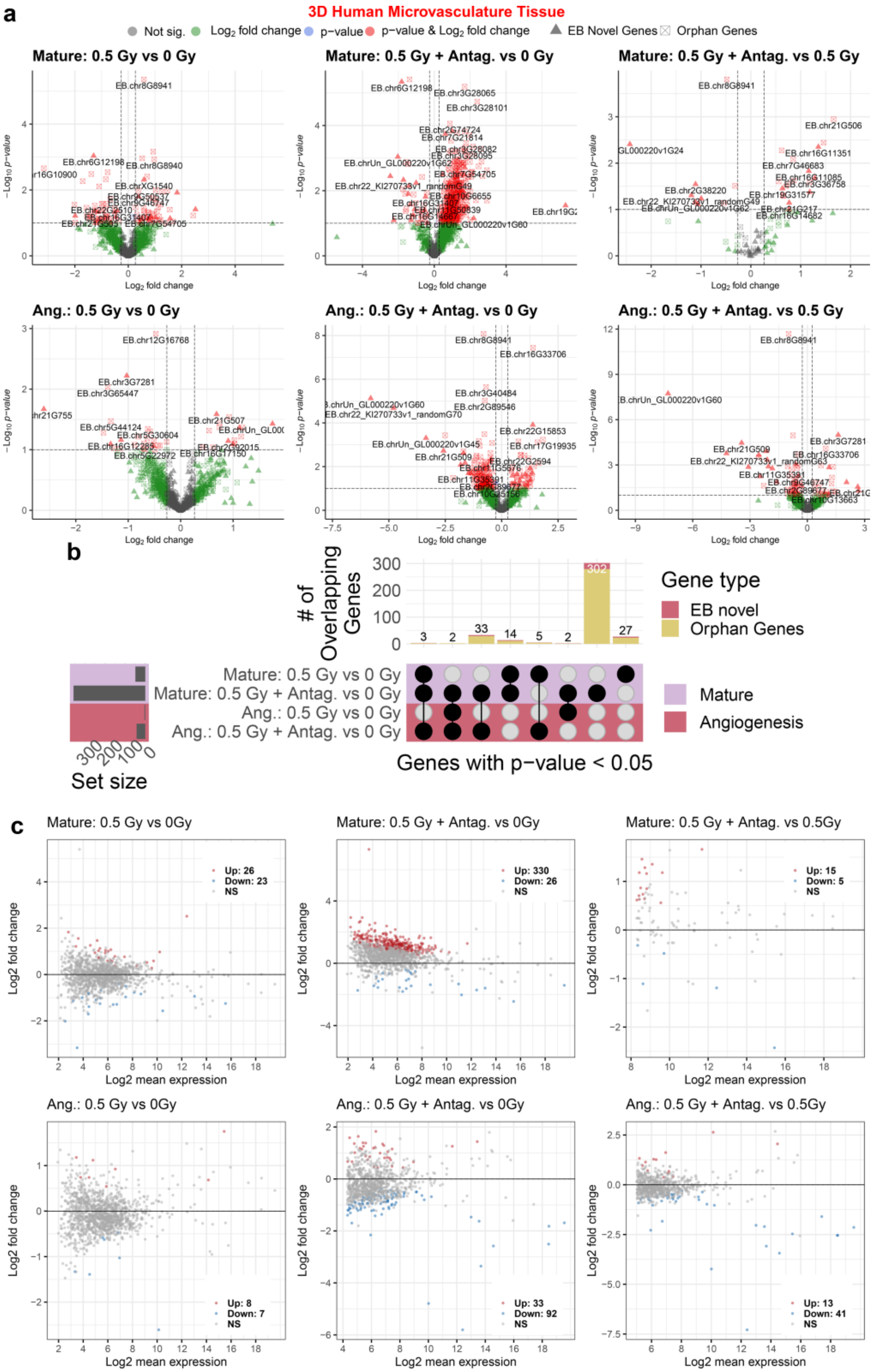

Supplementary Figure 4. Orphan and EB gene analysis on the RNA-seq analysis from the 3D mature and angiogenesis cell culture models. a) Volcano plots for the EB and orphan genes for each comparison. **b)** Upset plot displaying the overlapping significantly regulated EB and orphan genes for mature and angiogenesis models with and without antagomir treatment. **c)** MA plots for each comparison for just the EB and orphan genes.

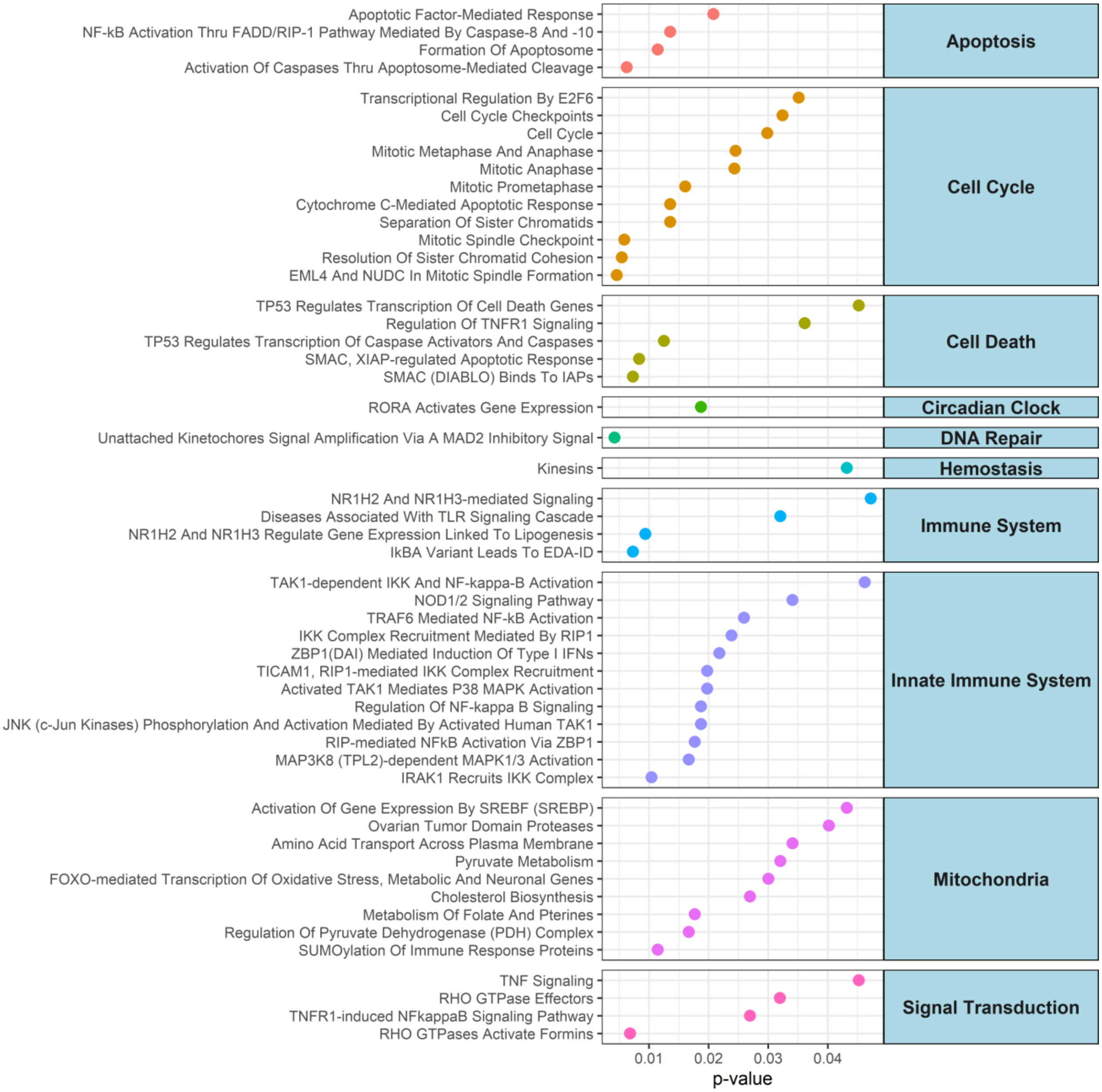

Supplementary Figure 5. Pathway analysis on the 21 key genes restored to basal level after antagomir treatment. Dot plots displaying the pathway analysis on the 21 genes from **Fig. 5** by the DAVID annotation tool.

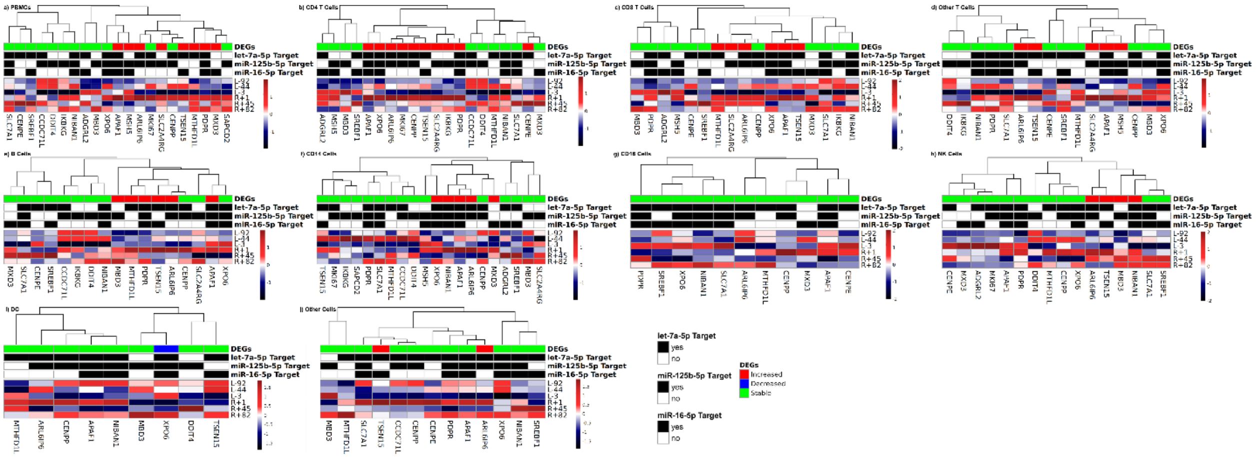

Supplementary Figure 6. Clustering of the 21 key genes from scRNA-sequence data from the i4 astronauts. **a)** - **j)** Clustered heatmaps displaying the 21 key gene profile for the different cell types (*i.e.* PBMCs, CD4 T cells, CD8 T cells, Other T cells, B cells, CD14 cells, CD16 cells, NK cells, Dendritic cells (DC), and other cells) from scRNA-seq on the i4 astronauts.

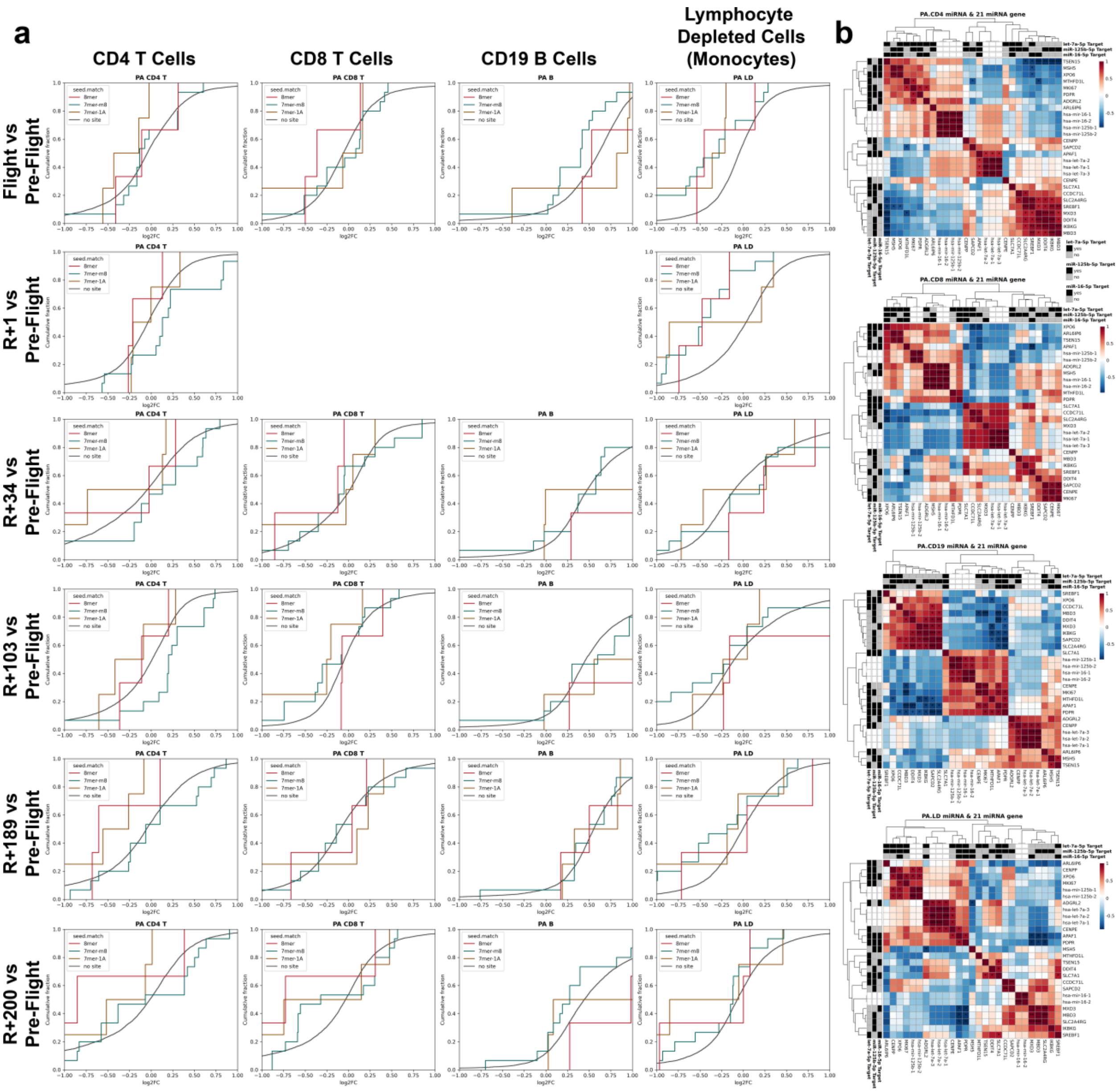

Supplementary Figure 7. The interaction of the three miRNAs with the 21 gene targets from the NASA Twin Study for all cell types. a) The change over time for the 21 genes targeted by the three miRNAs from the NASA Twin Study data for all cell types. Cumulative plots for the 21 key genes on RNA-seq data on CD4 T cells, CD8 T cells, CD19 B cells, and lymphocyte depleted cells (i.e. monocytes) from the NASA Twin Study from the Twin that was on the ISS for 340 days. There are comparisons over time starting at Flight vs Pre-Flight then comparing the different Return (R) to Earth timepoints starting at 1 day up to 200 days to Pre-Flight. **b)** Correlation plot of the three miRNAs (i.e. miR-16-5p, let-7a-5p, and miR-125b-5p) and the 21 gene targets for the overlapping time points per each sample type from the miRNA-seq and mRNA-seq NASA Twin Study data. The gene targets for the miRNAs are indicated in the outer black (yes for gene target) and gray (no for gene target) rows and columns. Significance for the correlation is shown by * = p-value < 0.05.

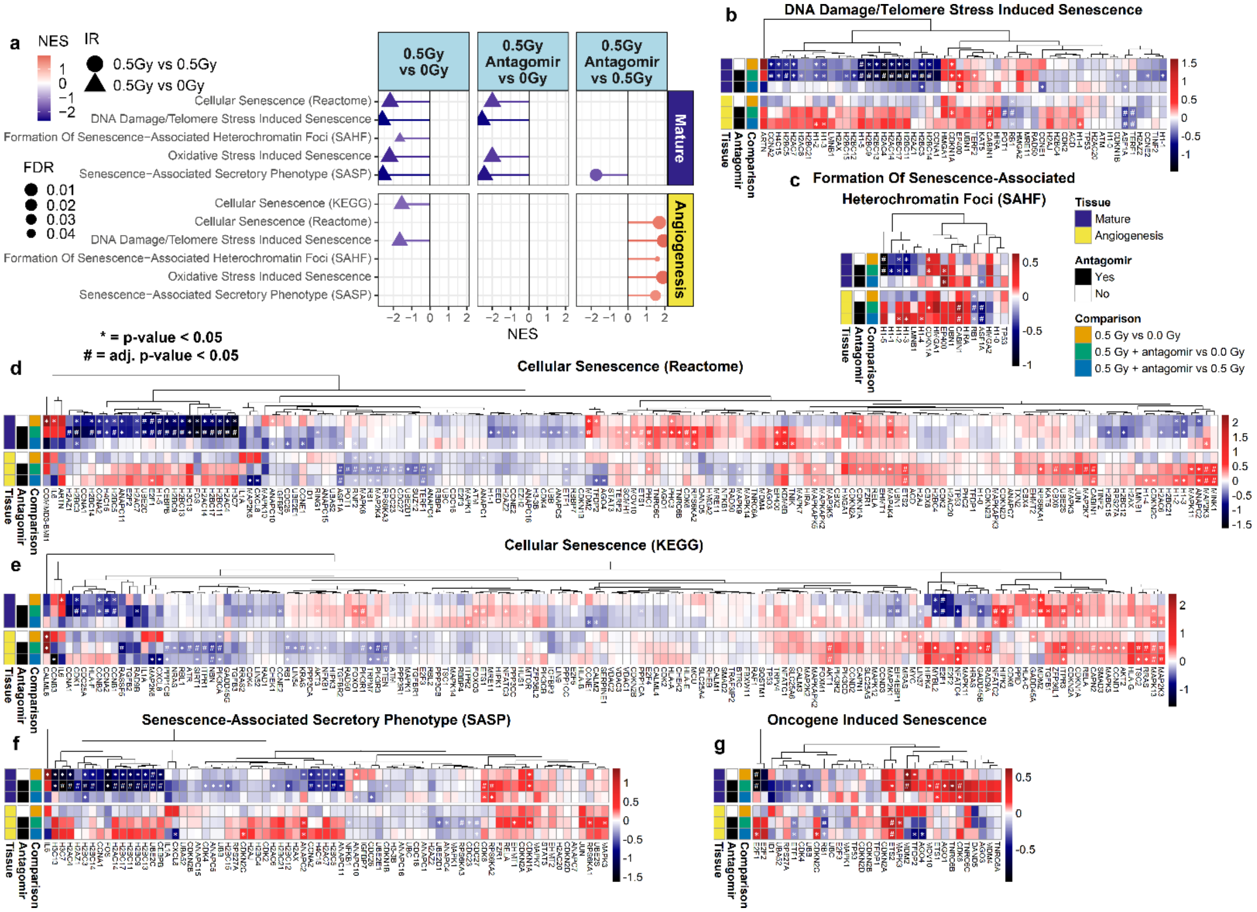

Supplementary Figure 8. Suppression of senescence related pathways and genes after irradiation with GCR. a) Lollipop plot of GSEA analysis on the custom senescence pathways for mature and angiogenesis cell cultures RNA-seq data. Only significantly regulated with pathways with FDR < 0.25 are shown. **b) - g)** Heatmaps of the t-scores for individual genes in the senescence pathways (*p-value < 0.05). For all RNA-seq data Wald test and the likelihood ratio test was used to generate the F statistic p-value.

Supplementary Table 1. Experimental validation for the gene targets to miR-16-5p, miR-125b-5p. and let-7a-5p from Fig. 5. The databases TarBase, miRTarBase, and starbase, provide literature and experimental references that demonstrate the proof that these miRNAs will target these genes. PMID = Pubdmed ID for the literature reference. For TarBase: Exp. = number of experimental evidence references, Pub. = number of publications demonstrating the validation, and Cell lines = number of validation done through cell line related experiments.

miR-16-5p									
**HGNC Gene ID**	**mirDIP Targets**	**# of Validated**	**PMID**	**TarBase v9.0**				**miRTarBase v9.0**	**starBase v2.0**
				**TarBase v9.0**	**Exp.**	**Pub.**	**Cell lines**		
**ADGRL2**	yes	2		Validated	14	10	8	No Evidence	Validated
**APAF1**	yes	2		Validated	8	8	5	No Evidence	Validated
**ARL6IP6**	no	1		Validated	2	1	2	No Evidence	No Evidence
**CCDC71L**	no	1		No Evidence				No Evidence	Validated
**CENPE**	no	1		Validated	2	2	2	No Evidence	No Evidence
**CENPP**	no	1		Validated	9	7	6	No Evidence	No Evidence
**DDIT4**	no	2		Validated	13	8	10	No Evidence	Validated
**IKBKG**	no	0		No Evidence				No Evidence	No Evidence
**MBD3**	no	1		Validated	2	2	2	No Evidence	No Evidence
**MKI67**	no	2		Validated	13	12	11	Validated	No Evidence
**MSH5**	yes	1		Validated	1	1	1	No Evidence	No Evidence
**MTHFD1L**	no	3	18668040	Validated	1	1	1	Validated	No Evidence
**MXD3**	yes	3	18668040	Validated	1	1	1	No Evidence	Validated
**NIBAN1**	yes	1		Validated	1	1	1	No Evidence	No Evidence
**PDPR**	yes	4	18668040	Validated	3	3	2	Validated	Validated
**SAPCD2**	no	1		Validated	4	4	4	No Evidence	No Evidence
**SLC2A4RG**	no	1		Validated	1	1	1	No Evidence	No Evidence
**SLC7A1**	yes	4	18668040	Validated	10	8	10	Validated	Validated
**SREBF1**	yes	1		Validated	7	6	6	No Evidence	No Evidence
**TSEN15**	yes	1		No Evidence				No Evidence	Validated
**XPO6**	yes	4	23622248	Validated	24	16	15	Validated	Validated

miR-125b-5p									
**HGNC Gene ID**	**mirDIP Targets**	**# of Validated**	**PMID**	**TarBase v9.0**				**miRTarBase v9.0**	**starBase v2.0**
				**TarBase v9.0**	**Exp.**	**Pub.**	**Cell lines**		
**ADGRL2**	no	0		No Evidence				No Evidence	No Evidence
**APAF1**	yes	1		No Evidence				No Evidence	Validated
**ARL6IP6**	yes	0		No Evidence				No Evidence	No Evidence
**CCDC71L**	yes	2		Validated	1	1	1	No Evidence	Validated
**CENPE**	no	0		No Evidence				No Evidence	No Evidence
**CENPP**	yes	2	20371350	No Evidence				Validated	No Evidence
**DDIT4**	yes	2		Validated	1	1	1	No Evidence	Validated
**IKBKG**	yes	0		No Evidence				No Evidence	No Evidence
**MBD3**	yes	0		No Evidence				No Evidence	No Evidence
**MKI67**	yes	1		Validated	2	2	2	No Evidence	No Evidence
**MSH5**	yes	0		No Evidence				No Evidence	No Evidence
**MTHFD1L**	no	0		No Evidence				No Evidence	No Evidence
**MXD3**	no	0		No Evidence				No Evidence	No Evidence
**NIBAN1**	yes	1		No Evidence				No Evidence	Validated
**PDPR**	yes	1		No Evidence				No Evidence	Validated
**SAPCD2**	yes	0		No Evidence				No Evidence	No Evidence
**SLC2A4RG**	yes	0		No Evidence				No Evidence	No Evidence
**SLC7A1**	yes	3	17891175	No Evidence				Validated	Validated
**SREBF1**	no	0		No Evidence				No Evidence	No Evidence
**TSEN15**	no	0		No Evidence				No Evidence	No Evidence
**XPO6**	yes	1		Validated	2	2	2	No Evidence	No Evidence

let-7a-5p									
**HGNC Gene ID**	**mirDIP Targets**	**# of Validated**	**PMID**	**TarBase v9.0**				**miRTarBase v9.0**	**starBase v2.0**
				**TarBase v9.0**	**Exp.**	**Pub.**	**Cell lines**		
**ADGRL2**	yes	1		Validated	18	13	11	No Evidence	No Evidence
**APAF1**	yes	1		Validated	19	11	11	No Evidence	No Evidence
**ARL6IP6**	yes	3	23622248	Validated	8	6	6	Validated	No Evidence

**CCDC71L**	yes	2		Validated	24	14	17	No Evidence	Validated
**CENPE**	yes	1		Validated	4	4	4	No Evidence	No Evidence
**CENPP**	yes	1		Validated	5	5	5	No Evidence	No Evidence
**DDIT4**	no	1		Validated	18	12	12	No Evidence	No Evidence
**IKBKG**	no	0		No Evidence				No Evidence	No Evidence
**MBD3**	no	1		Validated	6	3	5	No Evidence	No Evidence
**MKI67**	yes	1		Validated	11	5	7	No Evidence	No Evidence
**MSH5**	no	1		Validated	2	2	2	No Evidence	No Evidence
**MTHFD1L**	yes	1		Validated	4	3	3	No Evidence	No Evidence
**MXD3**	no	0		No Evidence				No Evidence	No Evidence
**NIBAN1**	yes	1		Validated	5	5	4	No Evidence	No Evidence
**PDPR**	yes	2		Validated	15	9	10	No Evidence	Validated
**SAPCD2**	no	1		Validated	1	1	1	No Evidence	No Evidence
**SLC2A4RG**	no	1		Validated	2	1	1	No Evidence	No Evidence
**SLC7A1**	yes	1		Validated	28	16	16	No Evidence	No Evidence
**SREBF1**	yes	1		Validated	6	5	5	No Evidence	No Evidence
**TSEN15**	yes	0		No Evidence				No Evidence	No Evidence
**XPO6**	yes	2		Validated	17	11	10	No Evidence	Validated
