# Supplementary material for: Space radiation damage rescued by inhibition of key spaceflight associated miRNAs
Source: Nat Commun. 2024 Jun 11;15:4825. doi: 10.1038/s41467-024-48920-y (PMC11166944; doi:10.1038/s41467-024-48920-y)
Supplement: Supplementary file 3 — Description of Additional Supplementary Information [file 41467_2024_48920_MOESM3_ESM.pdf]

Supplementary Information

Space Radiation Damage Rescued by Inhibition of Key Spaceflight Associated miRNAs

J. Tyson McDonald, JangKeun Kim, Lily Farmerie, Meghan L. Johnson, Nidia S. Trovao, Shehbeel Arif, Keith Siew, Sergey Tsoy, Yaron Bram, Jiwoon Park, Eliah Overbey, Krista Ryon, Jeffrey Haltom, Urminder Singh, Francisco J. Enguita, Victoria Zaksas, Joseph W. Guarnieri, Michael Topper, Douglas C. Wallace, Cem Meydan, Stephen Baylin, Robert Meller, Masafumi Muratani, D. Marshall Porterfield, Brett Kaufman, Marcelo A. Mori, Stephen B. Walsh, Dominique Sigaudo-Roussel, Mebarek Saida, Massimo Bottini, Christophe A. Marquette, Eve Syrkin Wurtele, Robert E. Schwartz, Diego Galeano, Christopher E. Mason, Peter Grabham, Afshin Beheshti

Supplemental Data 1. TPM values for all genes and conditions
Supplemental Data 2. TPM values for the 21 gene targets for the three miRNAs and conditions
Supplemental Data 3. All Gene Set Enrichment Analysis (GSEA) results for all pathways analyzed.
