# Supplementary material for: Space radiation damage rescued by inhibition of key spaceflight associated miRNAs
Source: Nat Commun. 2024 Jun 11;15:4825. doi: 10.1038/s41467-024-48920-y (PMC11166944; doi:10.1038/s41467-024-48920-y)
Supplement: Supplementary file 7 — Reporting Summary [file 41467_2024_48920_MOESM7_ESM.pdf]

nature portfolio

Corresponding author(s): Afshin Beheshti
Last updated by author(s): Apr 24, 2024

Reporting Summary

Nature Portfolio wishes to improve the reproducibility of the work that we publish. This form provides structure for consistency and transparency in reporting. For further information on Nature Portfolio policies, see our Editorial Policies and the Editorial Policy Checklist.

Statistics

For all statistical analyses, confirm that the following items are present in the figure legend, table legend, main text, or Methods section.

n/a	Confirmed	
☐	☒	The exact sample size (*n*) for each experimental group/condition, given as a discrete number and unit of measurement
☐	☒	A statement on whether measurements were taken from distinct samples or whether the same sample was measured repeatedly
☐	☒	The statistical test(s) used AND whether they are one- or two-sided<br>*Only common tests should be described solely by name; describe more complex techniques in the Methods section.*
☒	☐	A description of all covariates tested
☐	☒	A description of any assumptions or corrections, such as tests of normality and adjustment for multiple comparisons
☐	☒	A full description of the statistical parameters including central tendency (e.g. means) or other basic estimates (e.g. regression coefficient) AND variation (e.g. standard deviation) or associated estimates of uncertainty (e.g. confidence intervals)
☐	☒	For null hypothesis testing, the test statistic (e.g. *F*, *t*, *r*) with confidence intervals, effect sizes, degrees of freedom and *P* value noted<br>*Give P values as exact values whenever suitable.*
☒	☐	For Bayesian analysis, information on the choice of priors and Markov chain Monte Carlo settings
☐	☒	For hierarchical and complex designs, identification of the appropriate level for tests and full reporting of outcomes
☒	☐	Estimates of effect sizes (e.g. Cohen's *d*, Pearson's *r*), indicating how they were calculated

Our web collection on statistics for biologists contains articles on many of the points above.

Software and code

Policy information about availability of computer code

Data collection	The RNA-seq data from the 3D microvessel cell model data and the JAXA CFE data are available via the NASA Open Science Data Repository's (OSDR)'s Biological Data Management Environment with accession numbers: OSD-577, DOI: 10.26030/rs3g-e189 and OSD-530, DOI: 10.26030/r2xr-h714. The murine miRNA-seq data was also deposited on NASA OSDR's with the following identifiers, for all heart tissue related data: OSD-334, DOI: 10.26030/cg2g-as49; for all liver tissue related data: OSD-335, DOI: 10.26030/72ke-1k67; for all soleus muscle related data: OSD-337, DOI: 10.26030/m73g-2477; and for all plasma related data: OSD-336, DOI: 10.26030/qasa-rr29. Deposited data from the sequencing data from the NASA Twin Study can be found on the NASA Life Sciences Data Archive (LSDA) and the accession code is not available due to privacy concerns. LSDA is the repository for all human and animal research data, including that associated with this study. LSDA has a public facing portal where data requests can be initiated100. The LSDA team provides the appropriate processes, tools, and secure infrastructure for archival of experimental data and dissemination while complying with applicable rules, regulations, policies, and procedures governing the management and archival of sensitive data and information. The LSDA team enables data and information dissemination to the public or to authorized personnel either by providing public access to information or via an approved request process for information and data from the LSDA in accordance with NASA Human Research Program and JSC Institutional Review Board direction.<br>.<br>No Software was used for the data collection
Data analysis	R packages were used for analysis and plotting the data in the paper.

For manuscripts utilizing custom algorithms or software that are central to the research but not yet described in published literature, software must be made available to editors and reviewers. We strongly encourage code deposition in a community repository (e.g. GitHub). See the Nature Portfolio guidelines for submitting code & software for further information.

Data

Policy information about availability of data

All manuscripts must include a data availability statement. This statement should provide the following information, where applicable:

- Accession codes, unique identifiers, or web links for publicly available datasets
- A description of any restrictions on data availability
- For clinical datasets or third party data, please ensure that the statement adheres to our policy

The RNA-seq data from the 3D microvessel cell model data and the JAXA CFE data are available via the NASA Open Science Data Repository's (OSDR)'s Biological Data Management Environment with accession numbers: OSD-577, DOI: 10.26030/rs3g-e189 and OSD-530, DOI: 10.26030/r2xr-h714. The murine miRNA-seq data was also deposited on NASA OSDR's with the following identifiers, for all heart tissue related data: OSD-334, DOI: 10.26030/cg2g-as49; for all liver tissue related data: OSD-335, DOI: 10.26030/72ke-1k67; for all soleus muscle related data: OSD-337, DOI: 10.26030/m73g-2477; and for all plasma related data: OSD-336, DOI: 10.26030/qasa-rr29. Deposited data from the sequencing data from the NASA Twin Study can be found on the NASA Life Sciences Data Archive (LSDA) and the accession code is not available due to privacy concerns. LSDA is the repository for all human and animal research data, including that associated with this study. LSDA has a public facing portal where data requests can be initiated. The LSDA team provides the appropriate processes, tools, and secure infrastructure for archival of experimental data and dissemination while complying with applicable rules, regulations, policies, and procedures governing the management and archival of sensitive data and information. The LSDA team enables data and information dissemination to the public or to authorized personnel either by providing public access to information or via an approved request process for information and data from the LSDA in accordance with NASA Human Research Program and JSC Institutional Review Board direction. The Inspiration4 data has been uploaded to two data repositories: the NASA Open Science Data Repository (osdr.nasa.gov; comprised of NASA GeneLab and the NASA Ames Life Sciences Data Archive [ALSDA]), and the TrialX database. Identifiers for publicly downloadable datasets in the OSDR are documented as follows:
1) Data can be visualized online through the SOMA Browser (https://epigenetics.weill.cornell.edu/apps/I4_Multiome/), the single-cell browser (https://soma.weill.cornell.edu/apps/I4_Multiome/), and the microbiome browser (https://soma.weill.cornell.edu/apps/I4_Microbiome/).
2) For the PBMC data the data is available with OSDR accession ID: OSD-570 and the following link: https://osdr.nasa.gov/bio/repo/data/studies/OSD-570/.

Research involving human participants, their data, or biological material

Policy information about studies with human participants or human data. See also policy information about sex, gender (identity/presentation), and sexual orientation and race, ethnicity and racism.

Reporting on sex and gender	We did not perform sex dependent analysis and did not report sex or gender.
Reporting on race, ethnicity, or other socially relevant groupings	We did not report race, ethnicity, or other socially relevant groupings analysis
Population characteristics	For the NASA Twin Study data there were two male participants are reports. For the Inspiration4 data there were two male and two female participants. No age genotype, past diagnosis, and treatment information was presented in this manuscript and is available.
Recruitment	No bias in recruitment and this does not apply since we studied the limited astronaut data available to us.
Ethics oversight	The procedure followed guidelines set by Health Insurance Portability and Accountability Act (HIPAA) and operated under Institutional Review Board (IRB) approved protocols and informed consent was obtained. Experiments were conducted in accordance with local regulations and with the approval of the IRB at the Weill Cornell Medicine (IRB #21-05023569).

Note that full information on the approval of the study protocol must also be provided in the manuscript.

Field-specific reporting

Please select the one below that is the best fit for your research. If you are not sure, read the appropriate sections before making your selection.

☒ Life sciences ☐ Behavioural & social sciences ☐ Ecological, evolutionary & environmental sciences

For a reference copy of the document with all sections, see nature.com/documents/nr-reporting-summary-flat.pdf

Life sciences study design

All studies must disclose on these points even when the disclosure is negative.

Sample size	Sample size was limited to the number of astronauts allowed per mission.
Data exclusions	No data was excluded.
Replication	All samples were replicated. For the human data, The Twin Study data was done once over a 340 day period of time on the ISS. The Inspiration4 mission was only done once in a 3 day period. For obvious reasons these missions cannot be repeated due the extreme costs of sending humans to space. For the mice data, there were n=10 per group for a total of n=80 mice. For the 3D tissues for RNA-seq we utilized

n=5 biological replicates. For the vessel quantification in figure 1 the following was used: for mature the following biological independent samples were used: n=17 for 0Gy, n=18 for 0.5Gy, n=17 for 0.5Gy + 3 antagomirs, n=17 for 0.5Gy + let-7a-5p antagomir, n=17 for 0.5Gy + miR-16-5p antagomir, and n=16 for 0.5Gy + miR-125b-5p antagomir. For angiogenesis the following biological independent samples were performed: n=18 for 0Gy, n=20 for 0.5Gy, n=14 for 0.5Gy + 3 antagomirs, n=11 for 0.5Gy + let-7a-5p antagomir, n=14 for 0.5Gy + miR-16-5p antagomir, and n=10 for 0.5Gy + miR-125b-5p antagomir. For the DNA DSB assays the following was used: n=12 field of views for 0Gy and n=19 field of views for both 0.5Gy and 0.5Gy + antagomirs.

Randomization

Randomization cannot be done. There are two sets of experimental conditions: 1) samples on the ground (i.e. Earth) and 2) samples flown to space (i.e. International Space Station or in orbit). The samples are then analyzed accordingly.

Blinding

Blinding was not possible for human data, since we obtained the data were from limited astronauts. For the RNA-seq data the analysis were processed blinded. Do analysis we can't perform this blinded since we will need to run the proper statistics and analysis and blinding isn't possible. The same statement applies to the mice experiments.

Reporting for specific materials, systems and methods

We require information from authors about some types of materials, experimental systems and methods used in many studies. Here, indicate whether each material, system or method listed is relevant to your study. If you are not sure if a list item applies to your research, read the appropriate section before selecting a response.

Materials & experimental systems

n/a	Involved in the study
☐	☒ Antibodies
☐	☒ Eukaryotic cell lines
☒	☐ Palaeontology and archaeology
☐	☒ Animals and other organisms
☒	☐ Clinical data
☒	☐ Dual use research of concern
☒	☐ Plants

Methods

n/a	Involved in the study
☒	☐ ChIP-seq
☒	☐ Flow cytometry
☒	☐ MRI-based neuroimaging

Antibodies

Antibodies used

53BP1 (Catalog #: NB100-304, Novus Biologicals, Littleton, CO, USA)

Validation

This Antibody has been validated in our previous publications, In addition from Novus Biologicals:
Read Publications using
NB100-304 in the following applications:
B/N 4 publications
ChIP 1 publication
Chemotaxis 5 publications
FISH 1 publication
FLOW 11 publications
IB 2 publications
ICC/IF 392 publications
IF 3 publications
IF-FISH 1 publication
IF/ICC 1 publication
IF/IHC 22 publications
IHC 9 publications
IHC-Fr 5 publications
IHC-P 28 publications
IP 9 publications
ISH 6 publications
In Vivo 3 publications
KD 5 publications
KO 1 publication
PLA 3 publications
WB 94 publications

Eukaryotic cell lines

Policy information about cell lines and Sex and Gender in Research

Cell line source(s)

Prior to culturing 3D microvessels, HUVEC (Lonza Inc, Allendale, NJ, USA), primary cells isolated from the vein of the umbilical cord were cultured as 2D monolayers in complete endothelial basal medium (EBM; Lonza Inc., Allendale, NJ, USA), containing EGM medium (serum free) supplemented with 2% fetal bovine serum (FBS), human epidermal growth factor, hydrocortisone, and bovine brain extract. Cells were kept in a humidified incubator (5% CO2, 95% air) and the medium was changed twice a week. All cells used for experiments were cells thawed from a fresh vial.

Authentication	HUVEC cells obtained from Lonza
Mycoplasma contamination	The cell lines tested negative for mycoplasma
Commonly misidentified lines (See ICLAC register)	N/A

Animals and other research organisms

Policy information about studies involving animals; ARRIVE guidelines recommended for reporting animal research, and Sex and Gender in Research

Laboratory animals	C57Bl/6J wildtype female mice. 15-week +/- 3-day old, C57Bl/6J wildtype female mice were purchased from Jackson Laboratories. Upon arrival to BNL, mice were quarantined and acclimated to a standard 12:12 hour light:dark cycle, with controlled temperature/ humidity for 1-week prior to cage acclimation. Food and water were given ad libitum, and standard bedding was changed once per week. The normally loaded (NL) mice were originally utilized in parallel experiments with hindlimb unloaded mice which were not reported in this manuscript. Mice were cage acclimated (n=10 mice per group; 2 mice per cage to maintain social interaction) 3-days prior to HU, followed by 14-days either normally loaded (NL) or hindlimb unloaded.
Wild animals	No wild animals were used in this study.
Reporting on sex	Only Female mice were used
Field-collected samples	No field collected samples were used in the study.
Ethics oversight	All experiments were approved by Brookhaven National Laboratory's (BNL) Institutional Animal Care and Use Committee (IACUC) (protocol number: 506) and all experiments were performed by trained personnel in AAALAC accredited animal facilities at BNL, while conforming to the U.S. National Institutes of Health Guide for the Care and Use of Laboratory Animals. All methods were carried out in accordance with the relevant guidelines and regulations and are reported in accordance with ARRIVE guidelines.

Note that full information on the approval of the study protocol must also be provided in the manuscript.

Plants

Seed stocks	N/A
Novel plant genotypes	N/A
Authentication	N/A
